# Endosalpingiosis of Axillary Lymph Nodes: A Rare Histopathologic Pitfall with Clinical Relevance for Breast Cancer Staging

**DOI:** 10.1155/2016/2856358

**Published:** 2016-03-21

**Authors:** Laila Nomani, Benjamin C. Calhoun, Charles V. Biscotti, Stephen R. Grobmyer, Charles D. Sturgis

**Affiliations:** ^1^Robert J. Tomsich Pathology and Laboratory Medicine Institute, Cleveland Clinic, Cleveland, OH 44195, USA; ^2^Section of Surgical Oncology, Cleveland Clinic, Cleveland, OH 44195, USA

## Abstract

Establishment of accurate axillary lymph node status is of essential importance in determining both prognosis and the potential need for adjuvant therapy in patients with invasive breast cancer. Axillary lymph node heterotopias can in some cases result in overdiagnosis of metastatic disease. Nodal endosalpingiosis is perhaps the least commonly reported type of axially lymph node heterotopia. We herein illustrate a case in which second opinion pathologic interpretation combined with ancillary immunohistochemical studies allowed for a specific diagnosis of axillary nodal müllerian-type inclusions, confirming ypN0 staging and resulting in appropriate disease management and prognostication.

## 1. Introduction

Axillary lymph node status is one of the most important prognostic indicators for patients with invasive breast carcinoma, and intraoperative pathologic examinations of sentinel lymph nodes are performed at many centers to accurately stage patients as well as help determine the need for completion axillary dissections [1–4]. Various benign epithelioid cellular mimics of metastatic breast carcinoma in lymph nodes have been reported. These include but are not necessarily limited to heterotopic benign mammary epithelium, displaced cutaneous adnexal epithelial structures, nevus cell clusters, squamoid inclusions, mesothelial cell rests, and, most recently, müllerian-type epithelial inclusions (endosalpingiosis) [5–18]. All of these entities can be overinterpreted as metastases involving sentinel lymph nodes on intraoperative studies and/or in final pathologic evaluations of permanent sections. Such overcalls can result in falsely elevated nodal staging. Overdiagnosis of heterotopias as metastases may result in incorrect prognostic information and can potentially lead to overtreatment with surgical, radiation, and systemic therapies. Accurate interpretations are of paramount importance as misinterpretations of microscopic nodal structures may result in disproportionately large treatment changes. The histopathologic changes of endosalpingiosis are relatively commonly encountered in pelvic lymph nodes of adult females undergoing staging for gynecologic malignancies; however, such changes are perhaps the least commonly encountered type of benign epithelial inclusion in axillary nodes of women with breast carcinoma [19–28]. We herein report a case of endosalpingiosis involving an axillary lymph node in an adult female with a known diagnosis of invasive ipsilateral breast carcinoma.

## 2. Case Presentation

The patient was a 70-year-old female with a known history of invasive ductal carcinoma of the left breast. Her primary breast tumor was diagnosed via histologic assessment of an image-guided core biopsy at another facility. The invasive carcinoma was reported to measure more than 5 cm (clinical stage T3) by imaging studies. She was advised to undergo preoperative neoadjuvant chemotherapy. Upon completion of her neoadjuvant therapy, 6 months after the time of her original core biopsy diagnosis of invasive disease, the patient underwent left mastectomy and sentinel lymph node biopsies at the outside facility. No residual carcinoma was identified in the mastectomy specimen (complete histologic response to neoadjuvant therapy/ypT0); however, glandular epithelial structures were identified within one of the sentinel lymph nodes, and the outside pathologist forwarded the slides and tissue blocks from the lymph nodes and the original diagnostic core biopsy sample to our center for expert extradepartmental consultation. The best diagnosis for the glandular elements in the lymph nodes was in question.

**Figure 1:**
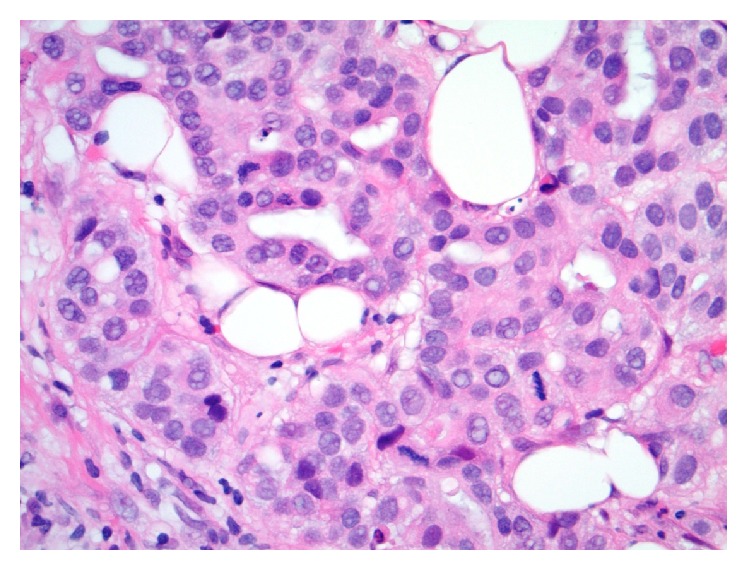
Photomicrograph of tissue section from the patient's known invasive ductal carcinoma, nuclear grade 2 (Hematoxylin and Eosin [H&E] stain, original magnification 400x).

**Figure 2:**
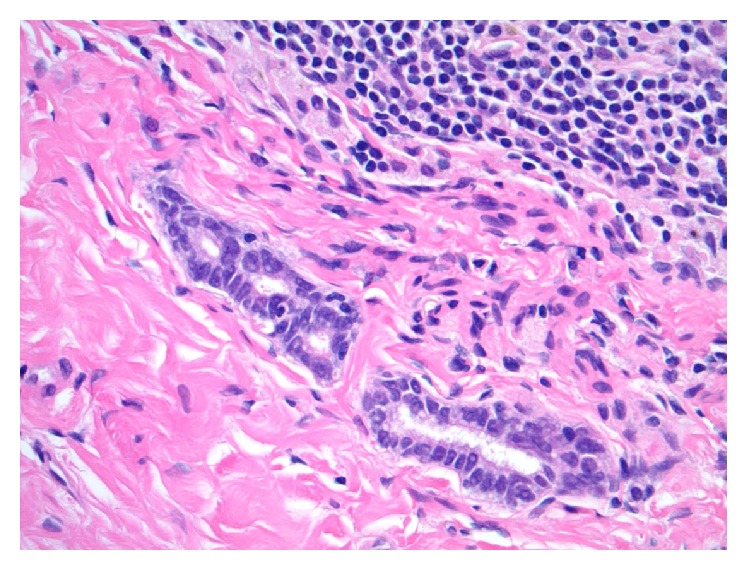
Photomicrograph of glandular epithelial structures within the capsular tissue of a postneoadjuvant, ipsilateral, and axillary sentinel lymph node (Hematoxylin and Eosin [H&E] stain, original magnification 400x).

The outside slides were reviewed, and the original core biopsy diagnosis of invasive ductal carcinoma, nuclear grade 2, was confirmed (Figure 1). The invasive tumor was 70% estrogen receptor positive by immunohistochemistry and was HER2 nonreactive/negative by IHC, scaled score 1+. The slides from one of the small, postneoadjuvant, excised sentinel lymph nodes demonstrated multiple (at least nine) distinct nests of glandular epithelium within the lymph node capsule (Figure 2). Some of these epithelial nests abutted the subcapsular sinus. These nodal capsular glandular cell groups were discontinuous and were seen to encircle approximately one-half of the circumference of the 3.5 mm lymph node. The histologic differential diagnosis for these glandular structures within the lymph node included metastatic disease versus benign/reactive heterotopic epithelium.

**Figure 3:**
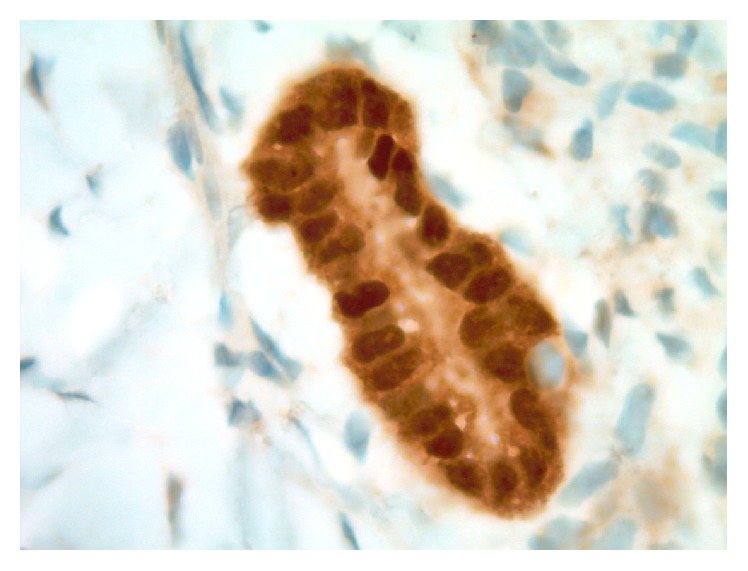
Photomicrograph of glandular epithelial rest in postneoadjuvant ipsilateral axillary sentinel lymph node capsule (PAX8 immunohistochemical study, original magnification 1000x).

**Figure 4:**
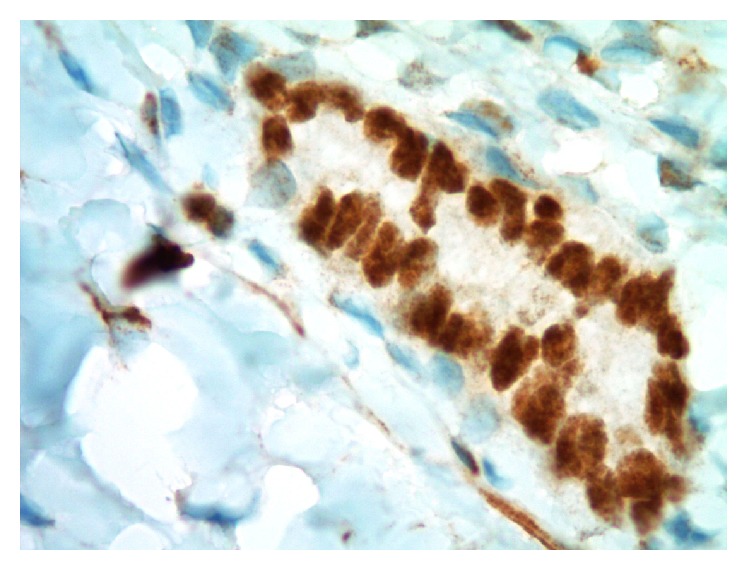
Photomicrograph of glandular epithelial rest in postneoadjuvant ipsilateral axillary sentinel lymph node capsule (WT1 immunohistochemical study, original magnification 1000x).

**Figure 5:**
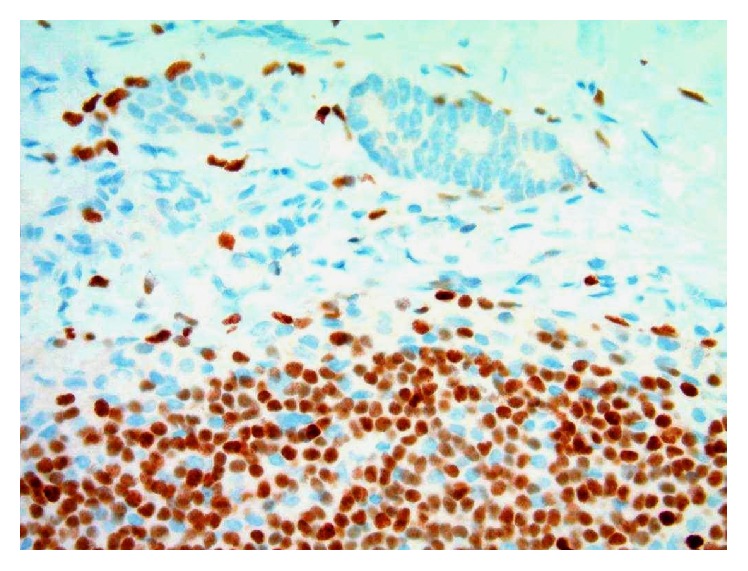
Photomicrograph of glandular epithelial rest in postneoadjuvant ipsilateral axillary sentinel lymph node (GATA3 immunohistochemical study, original magnification 400x).

**Figure 6:**
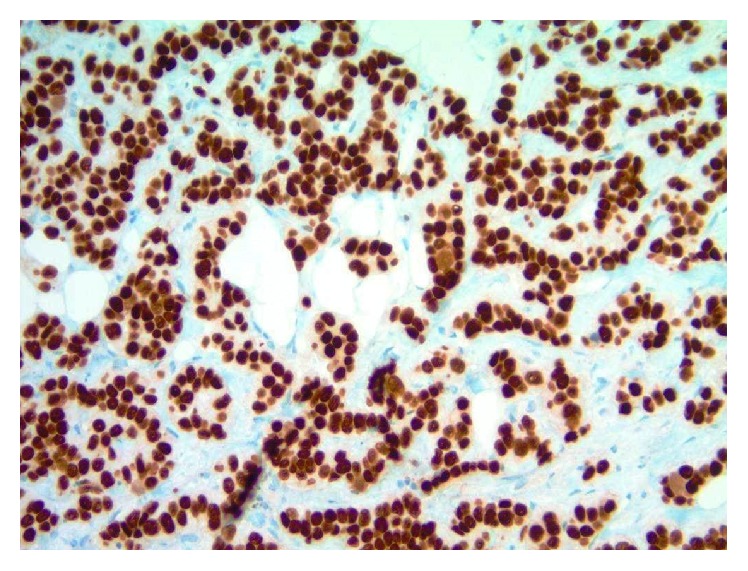
Photomicrograph of tissue section from the patient's known invasive ductal carcinoma (GATA3 immunohistochemistry, original magnification 200x).

**Figure 7:**
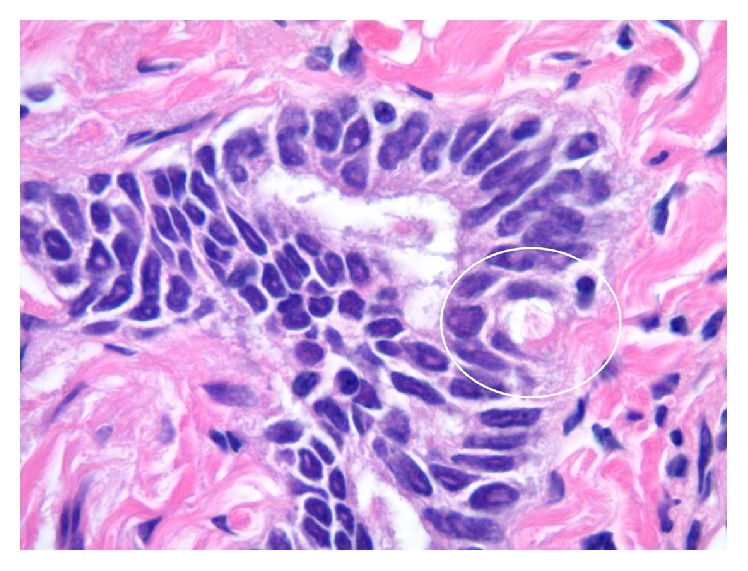
Photomicrograph of endosalpingiotic glandular rest in postneoadjuvant ipsilateral sentinel lymph node capsule with single cell ciliation and eosinophilic terminal bar circled in white (Hematoxylin and Eosin [H&E] stain, original magnification 1000x).

Histomorphologic comparisons between the capsular-associated glandular structures in the node and the patient's primary breast carcinoma core biopsy slides confirmed disparate appearances. The primary tumor showed greater nuclear pleomorphism and less overt tubule formation. In addition, the primary tumor was mitotically active, and no mitoses were seen in the nodal capsular epithelioid nests. Ancillary immunohistochemical (IHC) studies performed in our laboratory confirmed that the glandular cell groups in the periphery of the patient's sentinel node were immunoreactive (positive) for PAX8 (Figure 3) and WT1 (Figure 4) and negative for GATA3 (Figure 5). Slides from the original diagnostic breast core biopsy block were also prepared in our laboratory, and the invasive tumor was found to be positive for GATA3 (Figure 6) and negative for PAX8 and WT1, confirming a pattern of protein expression opposite of the glandular structures in the sentinel lymph node capsule. On 100x oil-immersion microscopy, rare singly scattered ciliated cells could be identified in the capsular nodal rests (Figure 7). A diagnosis of nodal endosalpingiosis (benign lymph node capsular müllerian inclusions) was rendered, and the patient's disease was then able to be accurately defined as ypT0, ypN0.

## 3. Discussion

On histologic examination, Müllerian-type glandular inclusions lined by cuboid to columnar epithelial cells that are reminiscent of fallopian tube type lining or coelomic type lining are commonly encountered within the capsules and/or peripheral cortices of pelvic lymph nodes from adult females. Case reports of endosalpingiosis specific to axillary lymph nodes are rare and have begun to appear within the literature in the last decade [22–26, 28]. The origins of gynecologic type epithelial and stromal heterotopias in lymph nodes are attributed by some to a theory of “müllerianosis” in which embryonic müllerian tissues that are displaced during organogenesis can result in the formation of at least four benign disease states, namely, developmental adenomyosis, endometriosis, endosalpingiosis, and endocervicosis [29]. In addition to a few previously reported cases of axillary nodal endosalpingiosis, one report of axillary nodal endocervicosis also exists [27]. All of the conditions included in the broader category of müllerianosis have been described as choristomal organoid structures composed of müllerian rests incorporated into other normal tissues/organs. All müllerian heterotopias are considered to be benign and can be definitively surgically managed [29]. The medical/scientific literature is uncertain as to whether or not endosalpingiosis can represent a precursor of malignancy. In addition to lymph nodes, heterotopic inclusions of tubal type epithelium have been reported in the peritoneal lining (by both biopsy and cytology), in the female reproductive organs including the cervix/uterus/ovaries, and in the urinary bladder, as well as in the soft tissues of the mediastinum and in the spleen [29–32]. A mouse model of morphologic ovarian endosalpingiosis exists with the describing authors suggesting that ovarian cortical epithelial cysts may be derived from the highly plastic ovarian surface epithelium resulting in endosalpingiosis-like ovarian inclusions [33].

The epithelial cells of endosalpingiosis are cytologically bland and are not seen in association with tissue invasion or associated desmoplasia. Ciliation, when present, is a reassuring feature, helping to confirm a benign diagnosis; however, many cases (such as our index case) present with a cuboidal cell morphology either completely lacking cilia or with only rare ciliated cells. Nonciliated forms of müllerianosis can be especially challenging to separate from metastatic adenocarcinomas, and in some instances endosalpingiosis may undergo hyperplastic changes making the distinction from malignancy even more difficult. In such cases, ancillary IHC testing may be of great value. While estrogen receptor (ER) IHC testing may be of value in differentiating metastatic breast carcinoma from some heterotopic rests, such as mesothelial rests or squamoid inclusions, ER is not a useful marker for distinguishing true mammary heterotopias and/or endosalpingiosis in lymph nodes, as these entities may also be ER positive. Certain commercially available IHC markers such as PAX8 and WT1, transcription factors/regulators involved in the development of the female reproductive system, are good markers of müllerian origin. Immunoreactivity (positivity) is commonly seen by IHC analysis in both fallopian tube epithelium and ovarian surface epithelium as well as in neoplastic proliferations derived from these cell types [31, 34–38]. These markers are typically nonreactive in breast epithelium and in neoplasms derived from breast epithelium. Alternatively, GATA3, a transcription factor important in breast luminal epithelial development, is immunoreactive by IHC studies in breast epithelium and in most tumors derived from breast epithelium [39, 40]. Some authors have shown sensitivities as high as 95% for GATA3 in labeling of mammary adenocarcinomas [41]. GATA3 IHC testing generally shows nonreactive (negative) results in müllerian epithelium and in epithelial lesions derived from the gynecologic tract. PAX8, WT1, and GATA3 IHC studies were of value in this case and helped to confirm the patient's ypN0 nodal status.

Pathologists should be aware that not all epithelioid glandular-appearing cell groups within lymph nodes and their capsules represent metastatic disease. Availing themselves of morphologic correlations between known primary tumor slides and subsequent nodal sampling slides is a good starting point when questions arise. IHC testing can be of great adjunctive value, and careful attention to cellular details, such as recognition of true ciliation, can prove immensely helpful. Seasoned breast clinicians who are the consumers of pathology reports and descriptions/images in multidisciplinary conferences should also be aware of axillary endosalpingiosis as an entity and should raise questions when appropriate. Unnecessary completion axillary lymph node dissections can be associated with arm movement disorders, chronic pain, paresthesias, lymphedema, and decreased quality of life. A correct diagnosis of axillary lymph node müllerianosis rests on a combination of awareness of existence and attention to light microscopic details. The goal of accurate classification is to avoid overdiagnosis of benign inclusions, müllerian and otherwise, so that staging and therapy are optimized for every patient.
